# eDNA based bycatch assessment in pelagic fish catches

**DOI:** 10.1038/s41598-024-52543-0

**Published:** 2024-02-05

**Authors:** Paulina Urban, Magnus Wulff Jacobsen, Dorte Bekkevold, Anders Nielsen, Marie Storr-Paulsen, Reindert Nijland, Einar Eg Nielsen

**Affiliations:** 1 Section for Marine Living Resources, National Institute of Aquatic Resources (DTU Aqua), Technical University of Denmark (DTU), Silkeborg, Denmark; 2 Section for Marine Living Resources, National Institute of Aquatic Resources (DTU Aqua), Technical University of Denmark (DTU), Lyngby, Denmark; 3 Section for Monitoring and Data, National Institute of Aquatic Resources (DTU Aqua), Technical University of Denmark (DTU), Lyngby, Denmark; 4 Marine Animal Ecology Group, Wageningen University, Wageningen, The Netherlands

**Keywords:** Marine biology, Molecular biology

## Abstract

Pelagic fish like herring, sardines, and mackerel constitute an essential and nutritious human food source globally. Their sustainable harvest is promoted by the application of precise, accurate, and cost-effective methods for estimating bycatch. Here, we experimentally test the new concept of using eDNA for quantitative bycatch assessment on the illustrative example of the Baltic Sea sprat fisheries with herring bycatch. We investigate the full pipeline from sampling of production water on vessels and in processing factories to the estimation of species weight fractions. Using a series of controlled mixture experiments, we demonstrate that the eDNA signal from production water shows a strong, seasonally consistent linear relationship with herring weight fractions, however, the relationship is influenced by the molecular method used (qPCR or metabarcoding). In four large sprat landings analyzed, despite examples of remarkable consistency between eDNA and visual reporting, estimates of herring bycatch biomass varied between the methods applied, with the eDNA-based estimates having the highest precision for all landings analyzed. The eDNA-based bycatch assessment method has the potential to improve the quality and cost effectiveness of bycatch assessment in large pelagic fisheries catches and in the long run lead to more sustainable management of pelagic fish as a precious marine resource.

## Introduction

Seafood plays a vital role in meeting the growing demand for nutrient-rich food and feed and thus it is crucial to future food supply^1^. The sustainable harvest of marine resources is challenging because of the poor condition of many fish stocks due to historical over-exploitation^2,3^, habitat destructive catch methods used^4^, and poor assessment of bycatch and discards^5^. Despite of being key-species in marine ecosystems, small, fast-growing pelagic species, such as herring, anchovies, sardines, and mackerel, are often high on the list of “eco-friendly” and "high nutritional value" fisheries^6,7^. The overall sustainability, however, is impeded by unintentional bycatch; defined as the accidental intake of non-target species^5,8,9^. Following the increase in public awareness on the issue of bycatch and discards in the early 2000s, increased focus has been put on identifying and estimating quantities of bycaught species^9–11^, and implementing rules and regulations such as the EU landing obligation^12^. Bycatch estimation is currently conducted using various approaches such as self-reporting by the fisherman via obligatory logbook documentation or through observer programs^9,13^. However, such methods can be biased as they rely on relatively rough visual estimates, the fisherman’s cooperation^14^, and manual species identification ease^15^. Only in a few countries, are fisher-independent (3rd party) estimates of bycatch gathered, which typically involve subsampling the catch and manually sorting and weighing the different species, the so-called “bucket method”, using relatively small sample size and frequency^16,17^. Basing bycatch estimates on relatively small samples is expected to be a particular problem for large catches of pelagic fishes, which commonly constitute more than 1000 tons in a single landing. Consequently, if the bycatch is heterogeneously distributed within the total landing, bycatch estimates can have high uncertainties if the number of buckets is low^18^. In addition, the estimates are commonly based on visual species identification and therefore labour intensive, costly, and possibly erroneous^15^ due to human error in relation to manual identification, sorting, and weighing. Hence, there are both qualitative and financial incentives to develop new technical solutions for catch composition analysis in pelagic fisheries.

In recent years, environmental DNA (eDNA) has become an effective and non-invasive tool for species monitoring in natural environments^19,20^. The basis of the concept is the release of DNA from all species into their surroundings through the shedding of skin cells, mucus, defecation, and other processes^21,22^. Subsequently, the pool of DNA can be collected from the environment (water, soil, air) and analysed using molecular methods. The two primary tools applied are quantitative PCR (qPCR) and DNA-metabarcoding. QPCR is a method for targeted identification of preselected species including precise DNA abundance estimation using species-specific primers^23^. The drawback is that only DNA from species targeted a priori can be evaluated. In contrast, DNA-metabarcoding can detect many different species simultaneously, through the use of universal primers^23^ that amplifies the DNA of many different species in a sample. The universality of DNA-metabarcoding comes at cost of quantitative DNA estimates^24^. This is primarily caused by “PCR selection”, whereby the match between the primer sequence and target species sequence leads to differential DNA amplification rates among species. Thus, the choice of method depends on the objective of the investigation, including the importance of quantitative versus qualitative results. Many applications of the eDNA concept to marine management and fisheries have been proposed and attempted, ranging from monitoring of economically profitable species^25–28^ to protected, endangered, and threatened species (PETS) in the wild^29,30^ or control and enforcement at fish auction houses and markets as means of battling illegal, unreported and unregulated fishing (IUU) fisheries^31^. Although the most focus of eDNA-based fisheries studies has been on qualitative aspects, quantitative species assessment has also been attempted, demonstrating correlations between abundance from traditional fish stock assessments and eDNA^32,33^. Likewise, monitoring of catch composition from the water surrounding the catch in trawl nets^34,35^ has shown correlations between species biomass and their DNA abundance. Recently, very strong quantitative relationships between fish weight and eDNA proportions were demonstrated in “production water” surrounding mixtures of pelagic fish^18,36^. These qPCR-based results demonstrate that sufficient precision, accuracy, and reproducibility can be attained in order to develop eDNA-based methods as a practical, validated tool for routine monitoring and control of large pelagic catches, ultimately resulting in a paradigm shift for catch monitoring. Although the results are promising, there is an urgent need for an end-to-end evaluation of eDNA-based catch assessment, from the choice of molecular tools over the experimental determination of eDNA/weight relationships to practical assessment of real fisheries samples. This includes an overarching evaluation of the reproducibility and robustness across the various steps of the process, as well as a detailed comparison of eDNA-based methods to the currently applied visual methods.

**Figure 1:**
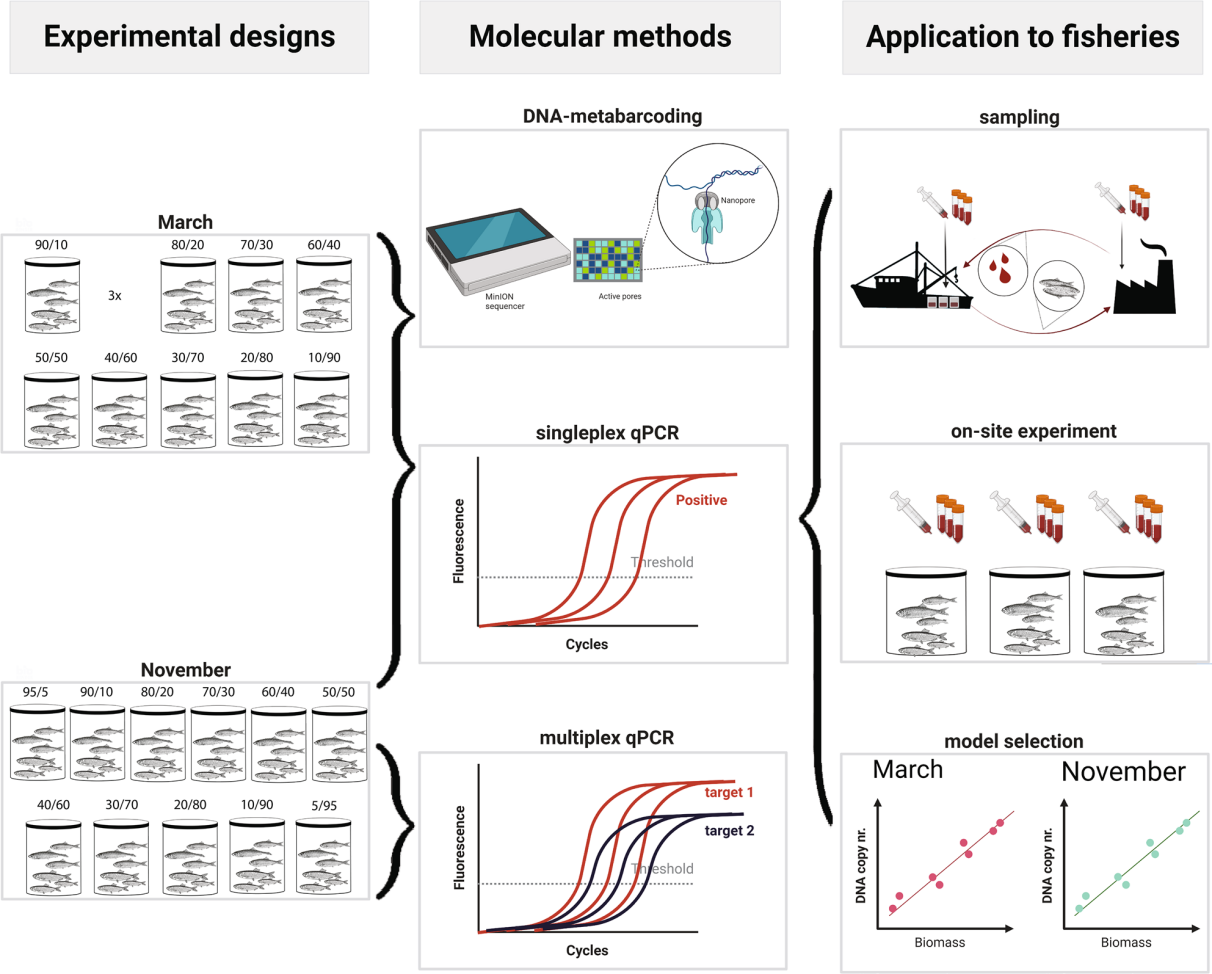
Outline of the study consisting of experimental eDNA to weight assessment, molecular method selection, and ground truth assessment through comparison to other bycatch assessment methods.

Here we describe and evaluate the full pipeline of the new concept of using eDNA based analysis for monitoring and controlling large pelagic fish catches. The concept relies on the basic assumption that DNA excreted from the catch to the surrounding production water is better mixed than the catch itself, thus heterogeneously distributed bycatch can be better represented by water than visually sorted and weighed subsamples collected at low size and frequency^18^. In the study we describe and critically evaluate the steps in the process including (a) sampling of production water for eDNA analysis at various stages in the catch-to-landing process, (b) choice of molecular tools, i.e., qPCR and metabarcoding, in relation to robustness, precision, and accuracy. (c) Establishment of relationships between DNA and fish weight, and (d) comparison of the eDNA-based outcomes to visually based bucket method applications, including fisherman’s logbook, 3rd party assessment, and fisheries control estimates (Fig. 1). As a representative model system for large pelagic fish catches, we use the sprat fishery in the Baltic Sea with substantial bycatch of herring to evaluate our concept. This simple catch-bycatch mixed fishery consisting of European sprat (*Sprattus sprattus*, L.) and Atlantic herring (*Clupea harengus*, L.), is complicated to resolve due to the high morphological similarity of the species and high heterogeneity in specimen distributions within catches^36^. The importance of assessing the herring bycatch is underpinned by the fact that the fishery is limited by the long-standing critical condition of the spawning stock biomass (SSB) of herring which has led to significant reductions in the allowed catch to help population recovery^37^. To assess the herring bycatch in the sprat fishery, we first collect samples of production water in fish storage tanks onboard fishing vessels (i.e. “ship production water”) and samples from the processing factory used to pump the fish from the vessel (i.e. “factory production water”) in order to evaluate the optimal point of sampling. Subsequently, we conduct controlled experiments with mock mixtures of sprat and herring with different known input weight fractions in order to establish a relationship between weight input and DNA output fractions for the two species. The controlled experiments simulate both production waters, at the ship and at the factory including repeated experiments for evaluation of potential seasonal effects, caused by changes in species condition (e.g., size or maturity stage) or changes in relation to temperature during fish landing (Table 1). The samples are subsequently analyzed using different molecular methods, species-specific singleplex and multiplex qPCR, and sequencing-based DNA-metabarcoding, to assess and evaluate their precision and accuracy in estimating herring weight/eDNA fractions. Based on the most robust molecular results we develop statistical eDNA-to-biomass models specific to each season and water type. We use on-site test samples prepared at each landing (consisting of 2–4 kg of catch with 30% of water) to select the adequate eDNA-to-biomass model. The most fitting models are then applied to eDNA samples from four different real sprat landings and the eDNA-derived estimates of herring bycatch are compared to visually-based estimates derived from logbook and catch subsampling (bucket method).

**Table 1:** Comparison of two experiments performed simulating the Baltic Sea sprat fisheries and aimed at establishing the eDNA-to-biomass relationship. Overall, the experiments show great similarities in the targeted mock samples, catchment area, and experimental facility. The main difference is the seasons studied and the temperature profiles (same temperature in ship production water experiments, however the November- experiment was shortly interrupted by a power outage, slightly lower temperatures during the factory production water experiment in November).

	March experiment	November experiment
Targeted mock samples, ratio of sprat to herring	90/10, 80/20, 70/30, 60/40, 50/50, 40/60, 30/70, 20/80, 10/90	95/5 90/10, 80/20, 70/30, 60/40, 50/50, 40/60, 30/70, 20/80, 10/90, 5/95
Season	March	November
Seawater take up	015°19′367 E 54°48′730 N	016°33′584 E 55°49′695 N
Seawater salinity	~ 10 PSU	~ 9 PSU
Catch place fish	015°35′111 E 54°36′775 N Bornholm Basin (South of Bornholm)	016°28′131 E 55°51′892 N Bornholm Basin (North of Bornholm, close to Öland)
Ship production water experiment duration (days)	8 days	7 days (power cut on day 6, increase in temp.)
Ship production water experiment place	RV DANA	RV DANA
Factory production water experiment duration	18 h	18 h
Factory production water experimental condition	6–7 °C, ~ 0 PSU	5–6 °C, ~ 0 PSU
Factory production water experiment place	10 min away from the ship landing place	2 h away from the ship landing place
Fish size	Large size difference between herring and sprat	Small size difference
Methods for DNA analysis	Singleplex qPCR, DNA-metabarcoding	Singleplex qPCR, Multiplex qPCR, DNA-metabarcoding

We provide an overall evaluation of the whole pipeline for eDNA-based estimation of bycatch and evaluate immediate prospects for implementation. We point to critical points in the process in order to develop rigid Standard Operating Procedures (SOPs) including an assessment of the eDNA-to-biomass model’s calibration frequency, guidance on model selection, and, finally, a cost-effectiveness assessment.

## Results and discussion

**Figure 2:**
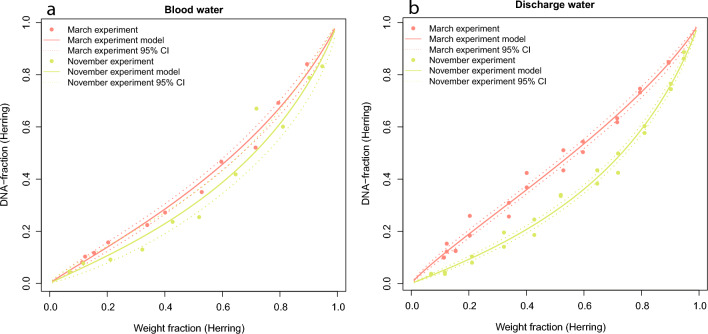
Comparison of the repeated experiments used to determine the eDNA-to-biomass relationship for ship production water (**a**) and factory production water (**b**). The solid line represents the model prediction and dotted lines the 95% confidence interval. Both experiments were prepared for fishing seasons relevant to the Baltic Sea sprat fisheries (March and November). In both water types, estimated herring DNA fraction was underrepresented in the November, compared to the March experiment.

The eDNA based analyses of the sprat/herring mock mixtures from the controlled experiments simulating production water showed strong linear relationships between estimated herring eDNA fractions and measured herring weight fractions. This was evident for both types of production water (ship and factory). The linear relationships were evident regardless of season (March/November). The eDNA fractions estimated from the controlled experiments simulating ship production water were almost identical (mean difference between eDNA fractions measured in March and eDNA fractions measured in November for the same mock mixtures being 0.047) (Fig. 2a), while there was a larger difference between the eDNA fractions in the simulated factory production water (mean difference of 0.121 in eDNA fractions for the same mock mixtures) (Fig. 2b). The systematic differences in eDNA fractions observed in factory production water between the two seasons could be caused by slight changes in the experimental setup (Table 1), temperature^36,38–41^, or natural variation in eDNA release rate per species^38,42^. The difference between the two experiments highlight the need for onsite calibration of the eDNA methods to increase the accuracy of the eDNA-to-biomass model for the fishery.

**Figure 3:**
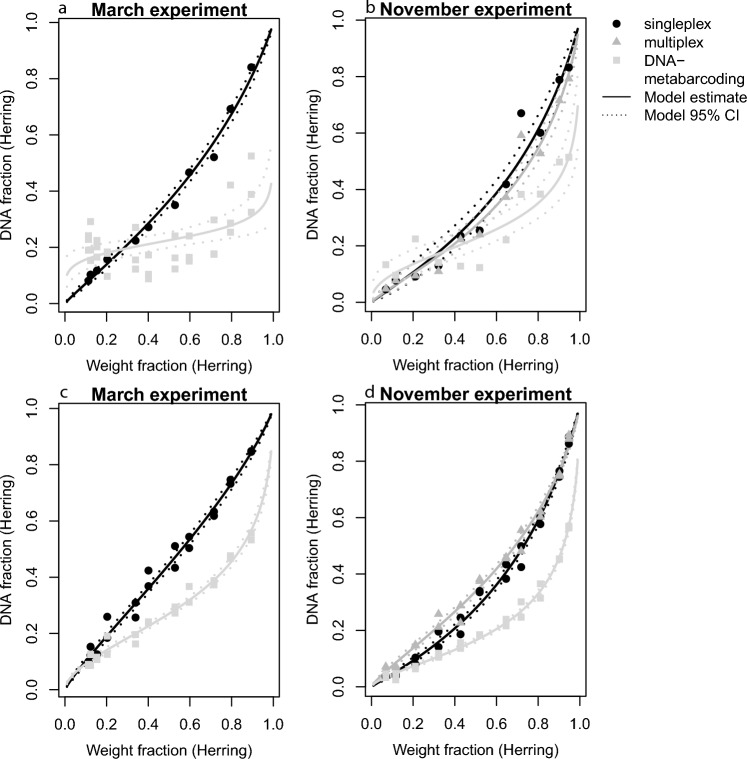
Comparison of the three molecular methods applied to the experimental samples (ship production water (**a**,**b**); factory production water (**c**,**d**)) for March (**a**,**c**) and November (**b**,**d**) experiment. Both qPCR based methods (singleplex, black dots, and multiplex, grey triangles) could be used interchangeably to derive accurate estimates of DNA fractions. The solid line represents the model prediction and dotted lines the 95% confidence interval. The DNA-metabarcoding approach, grey squares, showed differences in performance between the two water types analyzed. It appears that reliable DNA fractions can be estimated from “eDNA at factory” samples only.

The species-specific qPCR-based molecular methods, singleplex and multiplex PCR, provided the most precise and accurate estimates of herring eDNA fractions, with a strong linear relationship to herring weight input fractions in the mock mixtures of the controlled experiments. Further, both qPCR approaches showed a strong repeatability with regards to the herring eDNA-fraction measured (repeated measures GLM, *p* = 0.260). Herring fractions estimated using DNA-metabarcoding were significantly different from both qPCR-based estimates (repeated measures GLM, *p* < 0.001). Herring sequences were generally strongly underrepresented compared to sprat, which was likely due to the difference in the matching of the primers and the DNA sequences of the species (Supplementary Fig. S2). For metabarcoding ship production water, there was no clear linear relationship between weight fractions and eDNA fractions (Fig. 3a,b), whereas for factory production water there was a somewhat linear increase (Fig. 3c-d). Moreover, DNA-metabarcoding replicates of ship production water show large variation in the estimated herring eDNA fractions (Fig. 3a). The low precision and accuracy of DNA-metabarcoding compared to qPCR result from the method’s universality, i.e., by using primers that bind to all fish DNA rather than a single species. In short, PCR selection (and PCR drift) hamper the reproducibility of DNA-metabarcoding results for quantitative analysis (for a detailed discussion of the technical aspects see Supplementary Discussion S12). Following the stronger weight/eDNA relationships and higher reproducibility of the qPCR method we used results from the singleplex species-specific qPCR to develop eDNA-to-biomass conversion models specific for the fishing season (March and November) and production water type (ship and factory production water).

**Figure 4:**
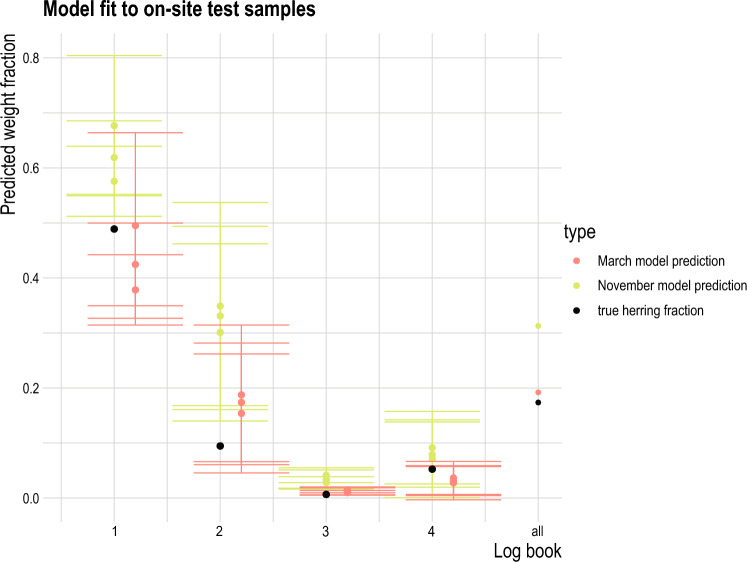
Shows the fit of each model March (pink) and November (green), to on-site test samples collected at each landing, and compares the predicted weight fraction to the true herring fraction found in the total on-site test sample (black). Each on-site test represented three replicates (each between 2–4 kg of the catch), that were first rinsed with freshwater and then sampled after 10 min, 2 h and 4 h. Each point in the graph represents the mean fraction for each time per landing and 95% confidence intervals across the three replicates. The weight numbers on top show the total amount of catch used for the on-site test sample.

The on-site test samples from the fishery generally showed good correspondence with the eDNA-to-biomass models derived from the controlled experiments (Fig. 4). For the ship production water samples, choosing between seasonal models did not affect the accuracy significantly, as models from both seasons provided very comparable results (Fig. 2 and Supplementary Fig. S4). For subsequent analysis of samples from fishing vessels we selected the March model, which was closest to the season when the real ship production water samples were collected (mid-January to mid-February). For the factory production water, the model developed based on the controlled experiment from March, was more compatible with the results from the on-site test samples, as it estimated herring weight fractions with higher accuracy than the model derived from the controlled experiment in November (Fig. 4, Supplementary Table S3). In landings 1 and 3 the March model clearly provided the most accurate results (Fig. 4), while for landing 2 both eDNA-to-biomass models appear to somewhat overestimate the herring weight fractions. In landing 4, the true weight fraction was between the two model estimates (Fig. 4). The discrepancy between the fractions estimated by the model and measured in the on-site test samples for the factory production water in landing 2 could be caused by the mature stage of the catch (17 days old) compared to “normal” catches that on average were landed after half the time at sea (6–10 days). Additionally, landing 2, unlike other landings and in the experimental setup, was not stored in seawater during the fishing process (hence no ship production water was generated). Since cooled production water at ship is used to preserve the catch during the fishing trip^43^, the fish could be more decomposed due to the long fishing duration and the poor storage of the catch, potentially skewing the eDNA-to-biomass relationship, although the relationship between eDNA and weight for mixtures of sprat and herring has been shown to be stabile over time^36^.

**Figure 5:**
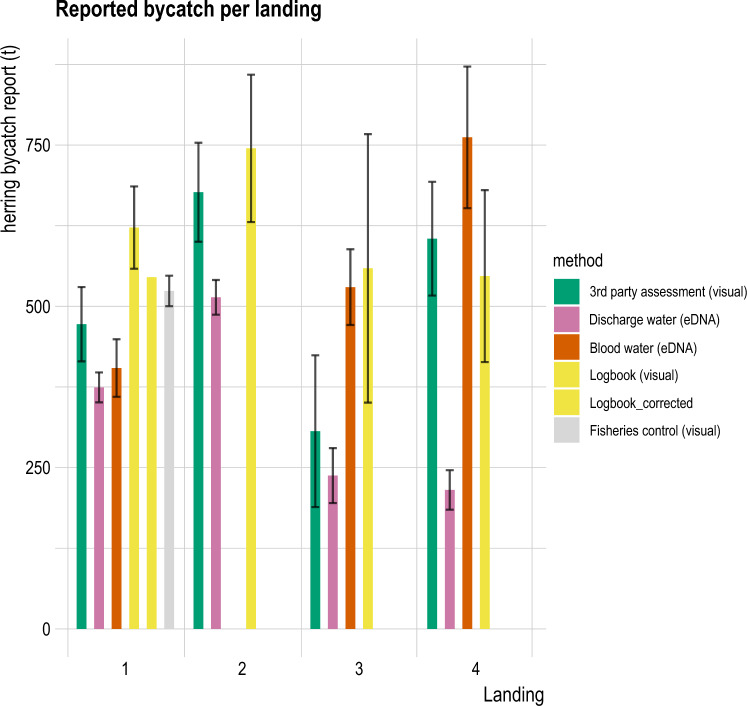
Overview of the herring bycatch estimated for each landing assessed using different methods. Estimates for landing 1 covered both eDNA based estimates; two different logbook estimates, the 3rd party assessment and additional estimated performed by the fisheries control agency that inspected the catch. For landing 2 no ship production water samples could be obtained, hence only factory production water. Error bars represent the standard error of the estimated bycatch rates per method.

**Figure 6:**
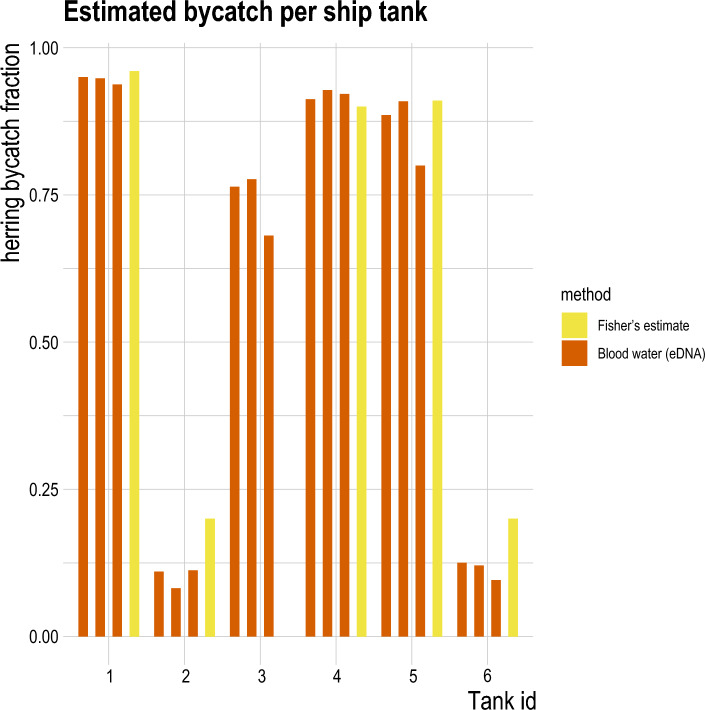
Detailed overview of the herring fractions estimated for each tank holding fish in landing 4. The eDNA estimates (orange) from ship production water are compared to additional fisher’s estimates (yellow). For all tanks, the estimates show substantial similarity.

For all landings, the eDNA-based method estimated the lowest herring bycatch biomass compared to the visual assessments using the bucket method (Fig. 5, Table 2), except for the ship production water samples in landing 4. The precision of eDNA based bycatch estimation was higher (lower standard error) than for the visual methods (Fig. 5). The eDNA based estimates were more precise for factory than ship eDNA samples. In landing 1, the estimates from the eDNA-based method differed the most from logbook and fisheries control derived bycatch estimates that both applied the bucket method for estimation (Fig. 5, Table 2). The eDNA-based method estimates were more similar to one another (the difference between ship and factory production water is 30 t), than the visual methods for the assessment, as 3rd party assessment and logbook estimates differed by 150 t, and 3rd party assessment, and fisheries control values differed by 52 t (Table 2). In landing 1, comparisons to the logbook should be done with care as two different logbook estimates were reported for this landing. In landing 2, the visual methods provided comparable results, with the difference between the 3rd party assessment and logbook estimates of 68 t. However, the standard error of both methods is very high (Table 2). In landing 3 and 4 the two eDNA-based estimates show quite deviating results (Fig. 5), with differences in estimates between ship and factory production water of 292 t in landing 3 and 547 t in landing 4 (Table 2). At both landings (3 and 4), the ship production water samples were collected before the last catch (haul) was made and not at the end of fishing as in landing 1 (Fig. 5), which very likely explains the discrepancy observed. Another factor for the observed difference could be the distribution of the bycatch rich hauls and bycatch poor hauls in separate tanks (Fig. 6, Supplementary Fig. S13). Accounting for the size of the tanks and mixing, i.e. the sequence in which the tanks are emptied to the factory, could level out both eDNA-based estimates^18^.

**Table 2:** Overview of estimates of bycatch per method for the four landings investigated in this study. Estimates from logbook, fisheries control and 3rd party assessment are derived following the visual estimation of bycatch from subsamples. For each method, we provide the end estimate of bycatch in kilograms (kg) of herring and as fraction of the total catch. Sample size for logbook assumed to be one estimate for haul, i.e. sample size equals amount of hauls.

Landing nr	Method	Herring bycatch estimate (t)	Herring bycatch standard error (t)	Herring bycatch estimate (fraction)	Sample size
1	Logbook	622.06	63.79	0.471	12
	Logbook_corr	545.25	NA	0.400	NA
	Fisheries control	523.89	23.60	0.385	63
	3rd party assessment	472.29	57.56	0.348	15
	Ship production water	404.35	44.65	0.297	5 (á 3 rep.)
	Factory production water	374.35	23.20	0.275	8 (á 3 rep.)
2	Logbook	744.88	114.25	0.492	18
	3rd party assessment	676.86	76.76	0.447	15
	Ship production water	NA	NA	NA	NA
	Factory production water	513.99	26.87	0.339	10 (á 3 rep.)
3	Logbook	558.86	208.11	0.388	6
	3rd party assessment	306.45	117.65	0.213	15
	Ship production water	529.79	58.69	0.368	8 (á 3 rep.)
	Factory production water	237.66	42.5	0.165	8 (á 3 rep.)
4	Logbook	546.89	133.26	0.441	10
	3rd party assessment	604.85	88.17	0.488	15
	Ship production water	761.96	109.78	0.614	6 (á 3 rep.)
	Factory production water	215.41	30.62	0.174	8 (á 3 rep.)

The total herring bycatch was unknown for all landings; thus the actual accuracy of methods cannot be evaluated. However, the relative precision of the eDNA-based method could be directly compared to the visually based bucket method. For landing 4, the fisher provided tank by tank herring bycatch estimates which enabled a more detailed comparison of the eDNA method’s accuracy for ship production water and logbook estimates (Fig. 6, Supplementary Table S11). The similarity between the fisher’s estimation and the eDNA-based estimates in individual tanks is striking. Not only are the visual and eDNA based estimates very similar, but the precision is also extremely high when comparing the three genetic replicates collected from each tank. The reason why the similarity in per-tank estimates of both methods does not translate directly into a high similarity for the overall estimate of herring bycatch in landing 4 is caused by lacking visual assessment for tank 3 in that landing. This illustrates the challenges regarding consistent logbook reporting and illustrates the potential for precise and accurate eDNA based bycatch assessment if a standardized eDNA sampling program is implemented.

## Conclusions

The case study of the full pipeline of eDNA based herring bycatch estimation in the sprat fishery illustrates that both production water types are a reliable and well-mixed source of DNA for large pelagic fish catches, which can be used for precise, and most likely accurate, bycatch quantification in fisheries. With the current available technology, it is apparent that qPCR-based solutions provide the best quantitative results. The eDNA-based method relies critically on information about how to translate eDNA into biomass. Thus, experimental establishment of robust eDNA/weight statistical models and frequent on-site model calibration for the translation of the species’ eDNA to biomass are essential for ensuring accurate results. The frequency and timing of calibration will depend on the specific properties of the fishery, such as the geographical location, and thus possible bycatch species, and seasonal spread of the fisheries, as many economically important pelagic fishes are fished during at least two seasons (i.e. Baltic Sea and North Sea sprat fisheries, North Sea herring fisheries^44^, and mackerel fisheries^45^). Accordingly, we recommend model selection and calibration at the start and at regular intervals during each fishing season. This can be achieved relatively easily through a simple, on-site mixture prepared from unloaded catches. This additional calibration step is likely to prove crucial to ensure accuracy and credibility of the method for monitoring control and enforcement. To further increase accuracy, accounting for size differences between fish species could be performed, as the size of species is known to influence the quantity of DNA released to the environment (i.e. allometric scaling)^36,46^ (Supplementary Fig. S12). In addition to the potential benefit regarding accuracy and precision when using eDNA for bycatch estimation compared to the visual bucket method, the eDNA based method is expected to halve the price of the bycatch estimation for the fishery. According to the Danish Pelagic Producers Organization the annual costs for the 3rd party assessment is 5–10 million DKK annually (covering 300–600 landings). For each landing, we propose that ca 30 eDNA samples must be taken and analyzed to ensure a trustworthy result. The cost for a DNA based analysis of 300 to 600 landings is then 2.25–4.5 million DKK at a price of 250 DKK per sample (price estimate per sample, including all steps from sampling to statistical analysis, provided by Eurofins Genomics, Europe Genotyping A/S), thus potentially halving costs for bycatch reporting. The cost for the eDNA-based analysis was prepared specifically for this study, where only two species were targeted for identification and quantification. In the case of a more diverse catch, the cost overview will shift directly proportional to the number of species which have to be analyzed with qPCR. Thus, with an increasing number of targets (i.e., species) for the analysis, price and time are expected to increase accordingly. Hence, for eDNA samples from more diverse fisheries other methods should be considered, such as droplet digital PCR which has a high precision and sensitivity and allows more targets (up to 12, Biorad QX600) to be analyzed simultaneously. In addition, we encourage more development of DNA-metabarcoding for quantitative applications, which could also provide an avenue for alleviating current limitations with regards to the number of species analyzed. Based on this and other studies, eDNA based bycatch quantification can likely be implemented in practical fisheries assessment within a short time period. Understandably, full evaluation of the applicability of the approach is limited by the small sample size used in this study. Thus, in order to fully grasp the scope of the method and facilitate its uptake by the relevant parties, it would be advisable to introduce the method alongside the traditional ways of estimating bycatch in fisheries in the near future. This would allow the fishermen, industry and authorities to further ground truth and familiarize with the new method. In the long run, improved estimates of bycatch quantities gained using the eDNA-based approach could improve the sustainability of pelagic fisheries-derived products such as fish meal and fish oil, through improved traceability of product composition, and quality of bycatch assessment models which would help to ensure exploitation of pelagic fish stocks within biologically safe limits and thus extensively contribute to sustainable pelagic fisheries worldwide.

## Materials and methods

### General description of the fishery process

A total catch from a single pelagic fishing trip typically results in landings at or above 1000 t^47^. To maximize quality the catch is kept in pre-cooled natural seawater in onboard tanks until landing^43^. This pre-cooled seawater is referred to as “ship production water”. Before the landing, hence the transfer of the catch from the fishing vessel to the processing factory on land the seawater is drained and replaced with freshwater. In contrast to the seawater, this freshwater (hereafter termed “factory production water”) is in contact with the catch for a relative short period (2–10 h), which could potentially have a different DNA composition than ship production water. In order to comply with the EU Landing Obligation, in Denmark the species composition of each sprat landing is estimated and reported by a logbook and by a 3rd party assessment^17^. Both methods rely on the “bucket method”, i.e. subsampling of the catch at different intervals, the identification and quantification of the species from the subsamples^18^. Additionally, during the landing process the total catch can be inspected by fisheries controllers that also use the bucket method on independent samples.

### Experiment: weight to eDNA relationship

The controlled experiments were prepared to mimic the Baltic Sea sprat fishery. The experimental units consisted different mock mixtures of sprat and herring prepared during the Baltic International Trawl Survey (BITS) on the research vessel DANA in March 2021^36^ and in November 2021 (Table 1). The experiments were conducted in almost exactly the same conditions. Table 1 presents a number of similarities and differences between the experimental set-ups.

Each mock mixture (i.e., experimental unit) in each experiment was subject to two different treatments (1) ship production water, (2) factory production water generation. The ship production water experiment was set up while at sea and water was sampled upon arrival at the harbour after the end of the cruise. Each experimental unit was given an ID and placed at random during treatments. Only the scientific crew responsible for setting up the experiment at sea and the lead scientists analyzing the data were aware of the ID allocation. The sampling consisted of collecting 45 mL water into sterile falcon tubes (Sarstedt, 50 mL), using a sterile syringe (Codan™, 60 mL). The falcon tubes were immediately frozen at -20 °C and kept until DNA-extraction. The mock mixtures (i.e. experimental units) were then transported to the lab, water and fish were separated using a mosquito net (mesh size: 1.4 × 1.6 mm) to avoid larger tissue particles. After draining, the fish were returned to the experimental containers double wrapped with new plastic bags (Cater Line, Freezer bags, 40 L). To simulate factory production water, 2 L of freshwater (tap water) was added to each of the experimental containers and stirred manually to ensure mixing. Plastic bags were closed to prevent possible cross-contamination and the containers were stored at 5–7 °C for the subsequent 18 h. The factory production water was sampled at two different time points: 2 h, and 18 h. All factory water samples were collected in 3 mL tubes (Sarstedt, 57 × 15.3 mm) using a sterile syringe (Injekt^®^, 20 mL). All units were stirred at regular intervals and before sampling, to ensure fully mixed samples. After collection, samples were frozen at − 20 °C until DNA-extraction.

### eDNA sampling of the pelagic sprat fishery

#### “On-site” test samples

In order to decide which model to use to most accurately translate the eDNA fractions to weight fractions at each landing we collected “on-site” test samples. Each sample consisted of three replicates of the catch (2–4 kg of catch), which were rinsed with freshwater three times before starting the experiment. The experiment started with pouring freshwater on top of the catch-subsample in defined proportion (30% of water for 70% of catch, i.e., approx. 300 mL of freshwater for 1 kg of subsampled catch). The mixture was thoroughly mixed and the factory production water consequently sampled at 10 min, 2 h and 4 h. The factory production water was sampled in triplicates, each replicate (up to 45 mL) was collected into a sterile 50 mL falcon tube (Sarstedt, Screw cap tube, 50 mL) using a sterile 60 mL syringe (Codan™). All samples were kept on ice during sampling and frozen at − 20 °C immediately after the end of the landing, until DNA-extraction. After water sampling the fish were visually identified and weight to determine the weight fraction in each replicate of the “on-site” test sample.

#### Sampling the fisheries

Production water samples from ships and factories were collected from four different landings of the sprat fishery in January–February 2021. All catches were harvested from different locations in the Baltic Sea (Supplementary Fig. S9). Each sampling started with collecting eDNA at ship samples from each holding tank (in total six tanks). Three replicates were collected from each holding tank (i.e., one sample = three replicates). Once the landing facility started discharging (landing of the catch from the ship to a land-based factory) we started with sampling the eDNA at factory samples, in triplicates at regular intervals (every 200t). We sampled up to 50 mL of production water from ship or factory into a sterile 50 mL falcon tube (Sarstedt, Screw cap tube, 50 mL) using a sterile 60 mL syringe (Codan™). All samples were kept on ice during sampling and frozen at − 20 °C immediately after the end of the landing. Logbook information and the 3rd party estimates for each landing were recorded.

### DNA-extraction

Before extraction, the samples were centrifuged at 3700 rpm for 30 s to minimize the chance of extracting tissue particles present in water. 1 mL of water was used for the extraction of DNA with the Omega Bio-tek E.Z.N.A. Tissue DNA kit (Omega Bio-tek, USA) following an adjusted version of their standard “tissue DNA protocol”, i.e., using a 2.5 × volume of buffers and solutions to adjust for the large sample volume. Samples were eluted in 50 µL pre-heated elution buffer, and stored at − 20 °C.

### Genetic analyses

#### Singleplex species-specific qPCR

Species-specific sprat^36^ and herring^28^ qPCR assays targeting the cytochrome b sequence of the mitochondrial DNA (mtDNA) were used for DNA quantification. Both assays were tested and validated in vitro in relation to assay optimization (primer and probe concentration adjustment), specificity (testing assay performance on closely related, co-occurring species and sensitivity with determination of LOD (Limit Of Detection) and LOQ (Limit Of Quantification)^36^. All samples were analyzed in duplicates on the StepOne Real-Time PCR System (Life Technologies, USA) with triplicate negative controls and triplicated dilution series ranging from 3 × 10^6^ to 3 × 10^0^ copies/reaction in each run. Total volume of each reaction was 10 µL with 3 µL of sample, 4 µL TaqMan™ Environmental Master Mix 2.0 (Thermo Fisher Scientific), assay-specific volumes of primers and probes to obtain optimal reaction conditions^36^ and 1.2 µL TaqMan™ Exogenous Internal Positive Control Reagents (Thermo Fisher Scientific) to monitor potential inhibition. The qPCR consisted of 5 °C for 5 min and 95 °C for 10 min followed by 50 cycles at 95 °C for 30 s and 60 °C for 1 min. Species-specific estimates of DNA copy numbers were then used to calculate herring and sprat fractions i.e. ratio of herring DNA copy number to total DNA copy number (sum of herring and sprat DNA copy) (‘DNA-based fractions’).

#### Multiplex species-specific qPCR

For the multiplexing qPCR the herring probe was modified TAMRA-dye to separate its fluorescence signal from the FAM-dyed sprat probe. An internal positive control (hereafter IPC) dyed with VIC was used in each reaction to monitor inhibition. We used the VetMAX Xeno Internal Positive Control containing BHQ-3 quencher (Applied Biosystems). The PCR settings for reagents and thermo cycler set up were the same as in the singleplex reaction (see above). Multiplexing was performed only on the November experimental samples (Table 1).

#### DNA-metabarcoding

The DNA-metabarcoding approach followed a 2-step PCR process. The first PCR amplified the Leray fragment (forward, mlCOIintF-ONT_fw : TTTCTGTTGGTGCTGATATTGC-GGWACWGGWTGAACWGTWTAYCCYCC^48^, reverse, jgHCO2198-ONT_rv: ACTTGCCTGTCGCTCTATCTTC-TANACYTCNGGRTGNCCRAARAAYCA)^49^. Each sample was PCR amplified using 10 µL 2 × Phire Tissue Direct PCR Master Mix (ThermoFisher Scientific, USA), 0.5 µL of each primer (10 nM), 2 µL template DNA and 7 µL DNA free water. The thermocycler settings consisted of initial denaturation at 98 °C for 180 s, 35 cycles of 98 °C for 10 s, 50 °C for 10 s, 72 °C for 20 s followed by a final extension at 72 °C for 180 s. The second PCR was used to attach a unique sample-tag to each sample that would allow sample pooling and sequencing on the same run. The second PCR was performed using the PCR barcoding kit 96 (PCB-096) (Oxford Nanopore Technologies Ltd., UK). We followed the manufacturer’s protocol, hence each sample was PCR amplified in total volume of 15 µL containing 12.5 µL LongAmp^®^ master mix (New England BioLabs^®^), 0.5 µL PCR barcode primer and 1 µL amplicon (from the first PCR) and 11 µL of DNA-free water. The cycling conditions consisted of initial denaturation at 95 °C for 180 s, 15 cycles of 95 °C for 15 s, 62 °C for 15 s, 65 °C for 90 s, and final extension at 65 °C for 180 s. Three replicates of the eDNA at ship samples collected from the March experiment were amplified and sequence separately to assess the effect of PCR amplification on the results. All PCR products were visually inspected on a 1% agarose gel. Sequencing was performed on a MinION Mk1C using R.10 flow cells and sequencing ligation kit SQK-LSK-112 (Oxford Nanopore Technologies Ltd., UK). All samples were pooled in equimolar rations prior to the library preparation step. All samples were run for 8 h, with a total amount of reads of 1.4 M for the first library (96 barcodes), and 2.5 M for the second library (36 barcodes). Raw reads were basecalled in Guppy (Version 6.1.1, Oxford Nanopore Technologies Ltd., UK), using super accuracy (SUP) mode. The performance of the runs (i.e. pore activity, pore availability, sequence length distribution) was visually inspected using Nanoplot (https://github.com/wdecoster/NanoPlot). The raw sequences were filtered to for lengths between 340 and 380 base pairs (bp) using decona (version 1.3, https://github.com/Saskia-Oosterbroek/decona). The FASTQ filtered files were then processed using Geneious Prime Software (Version 2021.2, Kearse et al. 2012). For each barcode, the sequences were classified against whole mtDNA genomes of herring and sprat. We chose a sequence overlap identity at min. 80% of 340 bp, from which 85% similarity was needed for sequence identification at species, 82% similarity for genus and 80% similarity at family level. The accuracy of the DNA-metabarcoding approach, including the settings for the taxonomic assignment, was tested on eleven mock samples of sprat/herring amplicons derived from the first PCR applied to tissue derived DNA (1 ng/µL) of each species respectively (95/5, 90/10, 80/20, 70/30, 60/40, 50/50, 40/60, 30/70, 20/80, 10/90, 5/95) (Supplementary Fig. S5). The DNA-metabarcoding approach was applied to experimental samples from both, March and November experiment (Table 1).

### Data analysis

Each experimental dataset served for establishing an eDNA-to-biomass model (one model per experiment, March or November, and method). For that beta- distributed generalized linear models (GLM) built using the package *glmmTMB* 1.0.2.9^50^ were used. The DNA quantities estimated from sprat and herring were converted into fractions; hence the genetic observations are continuous numbers between 0 and 1, naturally described by a beta distribution:

$$\begin{aligned}& O_{i} \sim {\text{Beta}}(\mu_{i} ,\;\upphi ), \hfill \\ & {\text{logit}}\;{(}\mu_{i} {) } = \, \upalpha + \upbeta \;{\text{logit}}({\text{true weight fraction}}_{i} ) \end{aligned}$$

O_i_ describes the observed DNA based fraction. O_i_ is assumed to be beta distributed with a mean value (⁠*µ*_*i*_) and a variance parameter (ϕ). The logit transformed mean value (⁠*µ*_*i*_) is assumed to be a linear function of the logit transformed true weight fraction.

In order to estimate herring biomass following eDNA fractions (biomass-to-eDNA) an inverse model of the above was used:

$${\text{weight}}\;{\text{fraction}}_{i} = {\text{ invlogit}}((O_{i} - \upalpha )/\upbeta )$$

To decide for the most suitable herring DNA fraction to biomass model for each landing, we used the “on-site” test samples prepared at each landing. We compared the weight estimates of each model to the true weight fractions derived from each “on-site” test sample.

Total bycatch per landing reported is the mean value of all sample replicates collected during each landing using each method (see sample size in Table 2). For the eDNA based estimates, eDNA fractions collected from multiple replicates were first translated into biomass fractions using the inverse model, and then a mean of all replicates per landing was calculated.

### Ethics statement

DTU has implemented the rules laid out in the EU-directive regarding animals in research (2010/63/EU – on the protection of animals used for scientific purposes). In Denmark, only invasive procedures, like surgical implants, chemical stress or behavioral studies with inflicted pain require specific permission by the National Board for Animals in research. The experiments were conducted on dead animals, hence, no animal welfare or animal use permits were required for this study, which is in accordance with local rules and regulations and fully comply with the DTU guidelines. The experiments followed the ARRIVE (Animal Research: Reporting on In Vivo Experiments) guideline. All molecular-based methods were performed following relevant local guidelines and regulations.

### Supplementary Information

Supplementary Information.

## Data Availability

The data from the eDNA-based method for the estimation of bycatch will be available online with a link provided after acceptance of the article https://github.com/12PU/Sprat-Fisheries-eDNA. Raw data from the logbook, the 3rd party assessment and fisheries control cannot be made public due to confidentiality agreements.
