# Supplementary material for: eDNA based bycatch assessment in pelagic fish catches
Source: Sci Rep. 2024 Feb 5;14:2976. doi: 10.1038/s41598-024-52543-0 (PMC10844201; doi:10.1038/s41598-024-52543-0)
Supplement: Supplementary file 1 — Supplementary Information. [file 41598_2024_52543_MOESM1_ESM.docx]

Supplementary Table S1 Overview of standard curve values of the singleplex and multiplex qPCR for the repeated November experiment (B and C) and for the sprat fishery data (A).

| A) Sprat fishery (singleplex) | | | | B) Repeated November experiment (singleplex) | | | | C) Repeated November experiment (multiplex) | | | |
| --- | --- | --- | --- | --- | --- | --- | --- | --- | --- | --- | --- |
|  |  |  |  |  |  |  |  |  |  |  |  |
| target species | R2 | Eff% |  | target species | R2 | Eff% |  | target species | R2 | Eff% |  |
| sprat | 0.998 | 102.35 |  | herring | 0.998 | 95.92 |  | sprat | 0.997 | 94.39 |  |
| herring | 0.996 | 94.62 |  | sprat | 0.998 | 95.35 |  | herring | 0.998 | 90.53 |  |
| sprat | 0.997 | 92.49 |  | herring | 0.996 | 92.90 |  | sprat | 0.997 | 91.12 |  |
| herring | 0.999 | 97.19 |  |  |  |  |  | herring | 0.996 | 99.22 |  |
| sprat | 0.999 | 97.65 |  |  |  |  |  |  |  |  |  |
| herring | 0.996 | 90.04 |  |  |  |  |  |  |  |  |  |
| sprat | 0.998 | 97.50 |  |  |  |  |  |  |  |  |  |
| herring | 0.997 | 91.34 |  |  |  |  |  |  |  |  |  |
| sprat | 0.993 | 100.12 |  |  |  |  |  |  |  |  |  |
| herring | 0.998 | 95.91 |  |  |  |  |  |  |  |  |  |
| sprat | 0.996 | 95.42 |  |  |  |  |  |  |  |  |  |
| herring | 0.997 | 93.54 |  |  |  |  |  |  |  |  |  |
| sprat | 0.997 | 90.42 |  |  |  |  |  |  |  |  |  |
| herring | 0.995 | 89.78 |  |  |  |  |  |  |  |  |  |
| herring | 0.994 | 93.89 |  |  |  |  |  |  |  |  |  |
| sprat | 0.996 | 93.24 |  |  |  |  |  |  |  |  |  |

Supplementary Figure S2 DNA-metabarcoding primer mismatch in sprat and herring mtDNA. Forward primer is the mlCOIintF with 1 mismatch for sprat and 4 mismatches in herring, the reverse primer jgHCO2198 matches both species perfectly.

Forward primer mlCOIintF (GGWACWGGWTGAACWGTWTAYCCYCC)


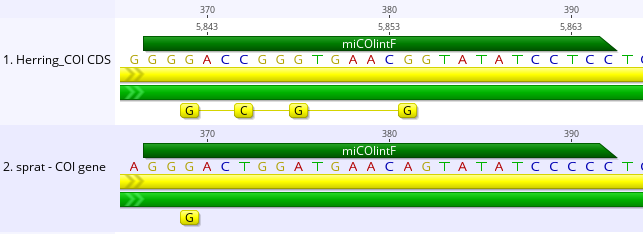


Reverse primer jgHCO2198 (TANACYTCNGGRTGNCCRAARAAYCA)


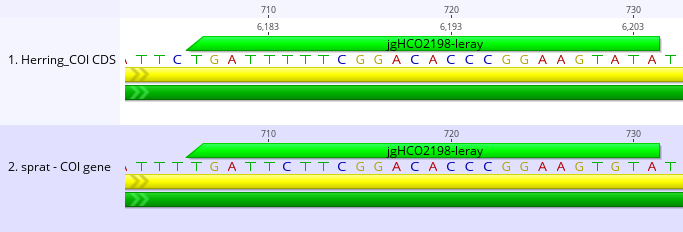


Supplementary Table S3 The accuracy in herring biomass prediction using the November experiment and March experiment on eDNA estimates derived from the on-site test samples collected at factories (hence discharge water).

|  | **variance** | **bias of the estimator** | **root mean square error** |
| --- | --- | --- | --- |
| March experiment prediction | 0.031337 | 0.000209 | 0.076097 |
| November experiment prediction | 0.031337 | 0.121018 | 0.159227 |


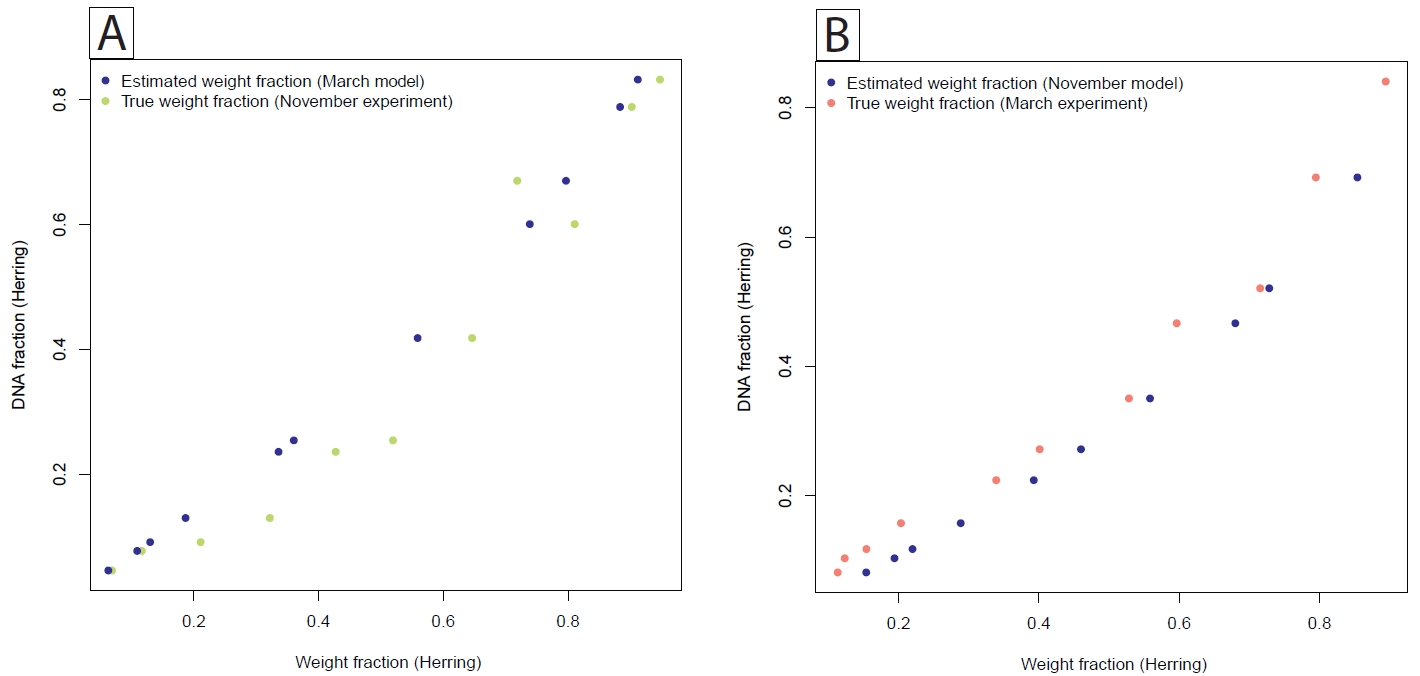
Supplementary Figure S4 Estimated vs. True measured fraction for the November (A) and the March (B) experiment based on the singleplex qPCR. For the estimation in A the model developed based on March sample was used, for B the one based on November samples. In both cases, the true and estimated fractions show very comparable results.


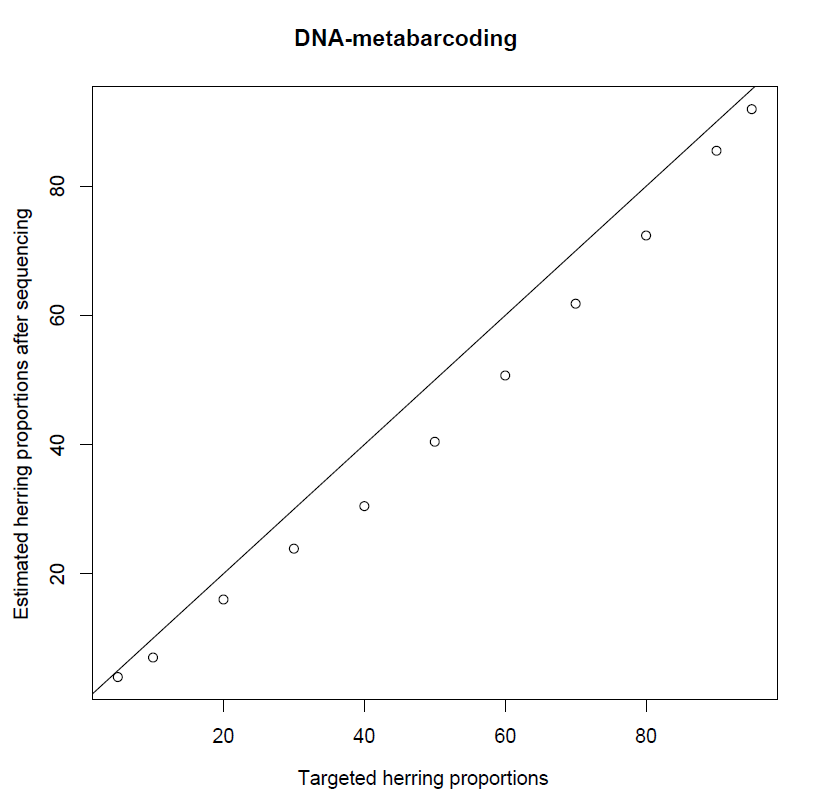


Supplementary Figure S5 Testing accuracy of the two-step DNA-metabarcoding approach. Eleven sprat-herring-amplicon mixtures with targeted herring proportions were prepared from sprat and herring amplicons (derived from the first PCR). These mixtures underwent the second PCR step, the barcoding PCR, in the same way as the experimental samples. The identification of reads was performed in Geneious (as described in material and methods). Estimated proportions from the sequencing run show a strong linear relationship to the targeted herring proportions (open circles). The solid line indicates the 1:1 straight line.

Supplementary Figure S6 DNA-metabarcoding results from the seawater blank samples collected from each experimental unit before starting the experiment. The seawater blanks were processed along the blood and discharge water samples. During the pooling of samples for library preparation all samples, including blanks, were pooled in equimolar concentrations.


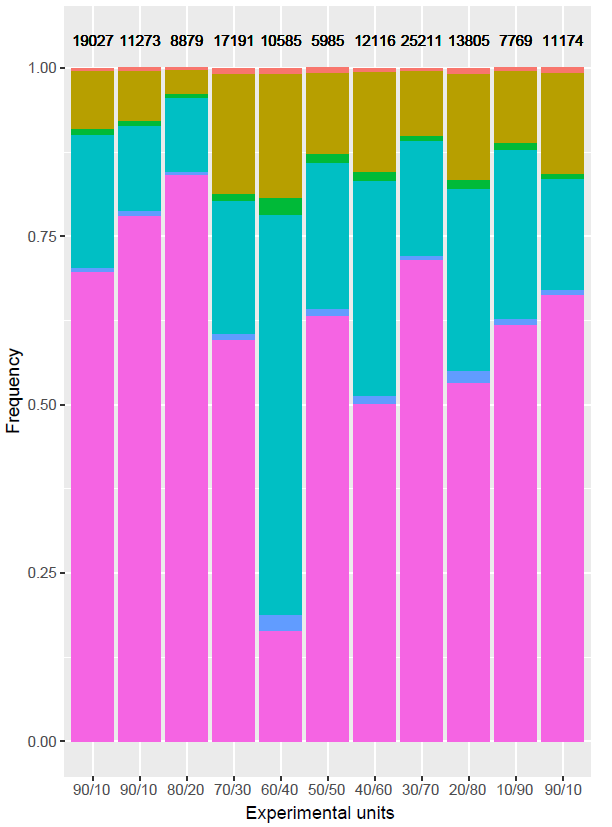

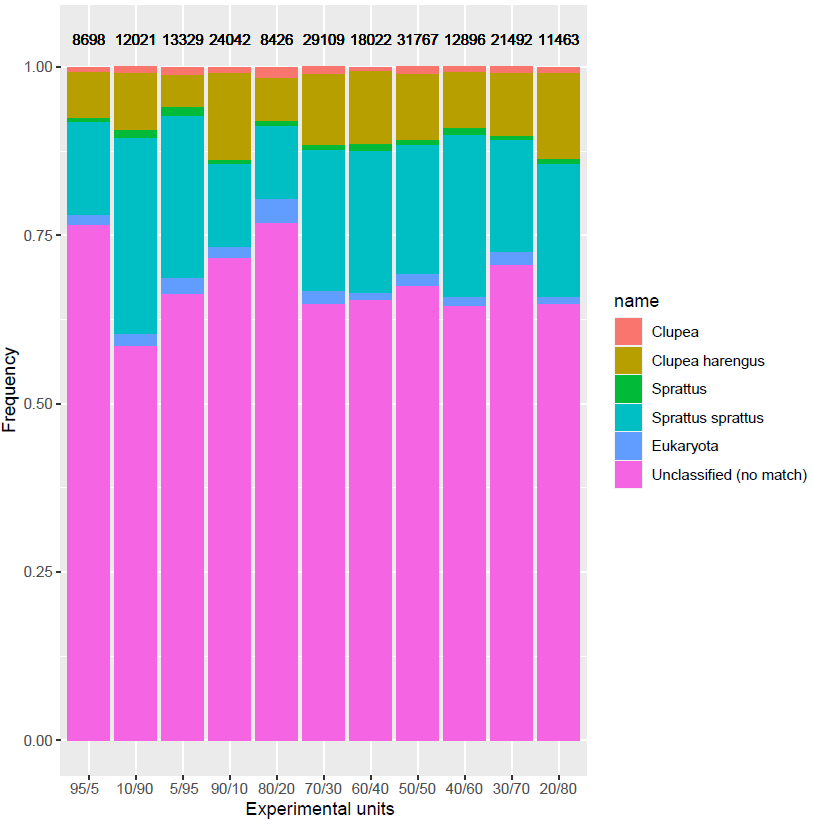


**A**

**B**

Supplementary Figure S7 DNA-metabarcoding results from the DNA extraction blanks collected during the DNA-extraction processed. The extraction blanks were processed along the blood and discharge water samples. During the pooling of samples for library preparation all samples, including blanks, were pooled in equimolar concentrations.


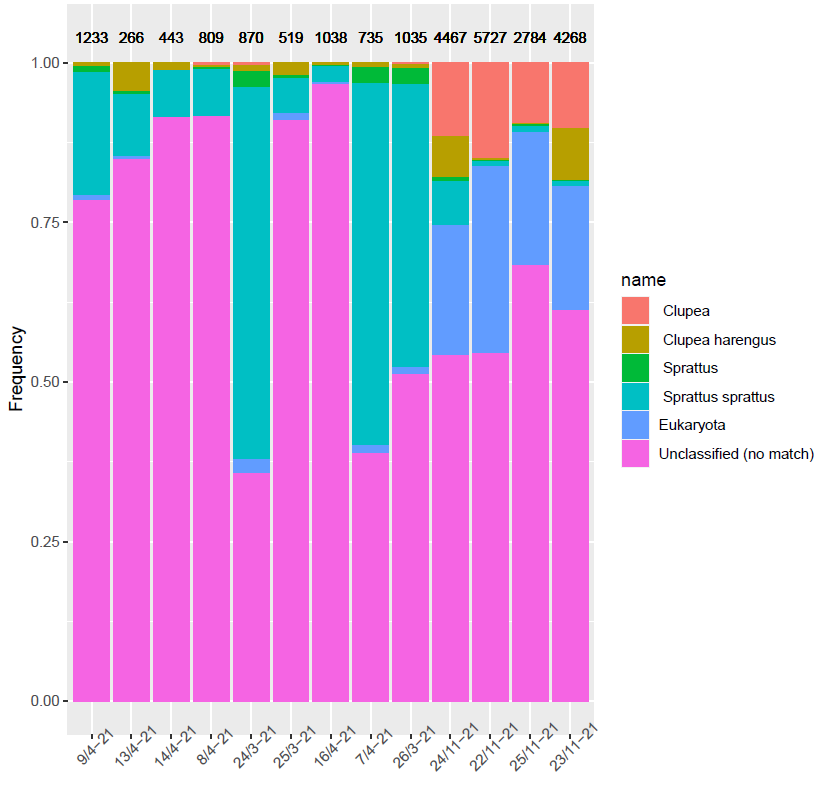


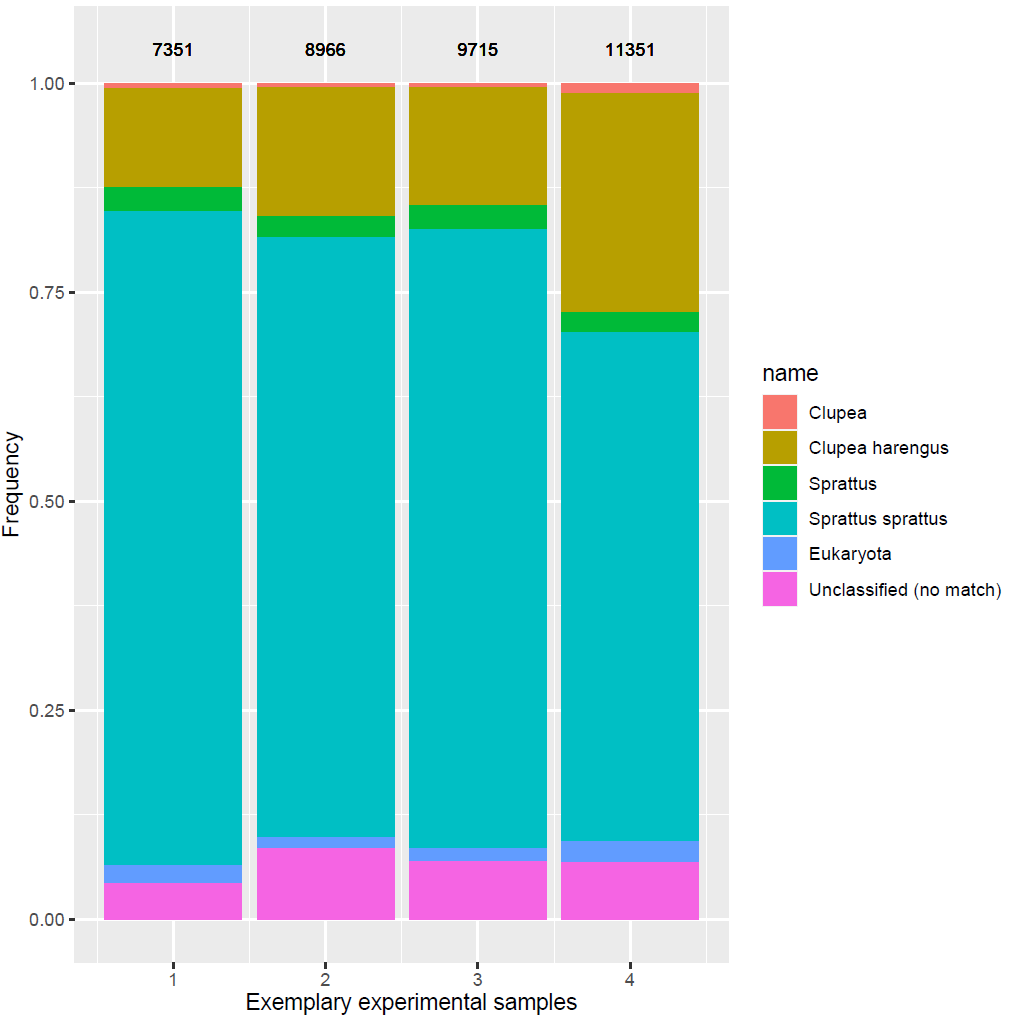
Supplementary Figure S8 Frequency of sequences assigned to different taxonomical levels using the settings described in the material and methods under genetic analyses (3. DNA-metabarcoding). The frequency is illustrated for four exemplary experimental samples analyzed. The value on top of the graph shows the total amount of sequences detected for each sample.

Supplementary Figure S9 Geographical distribution of the 4 total catches (landings) of the Baltic Sea sprat fisheries investigated in this study. All catches were made in the winter fishing season for the fishery (between January and beginning of March).


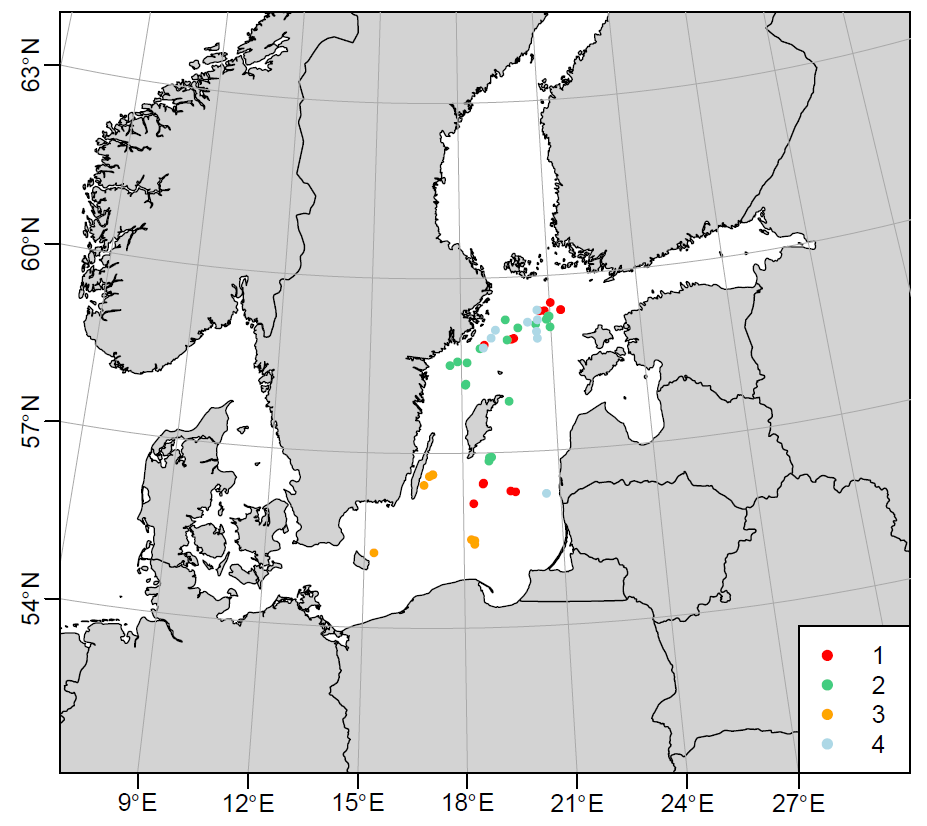


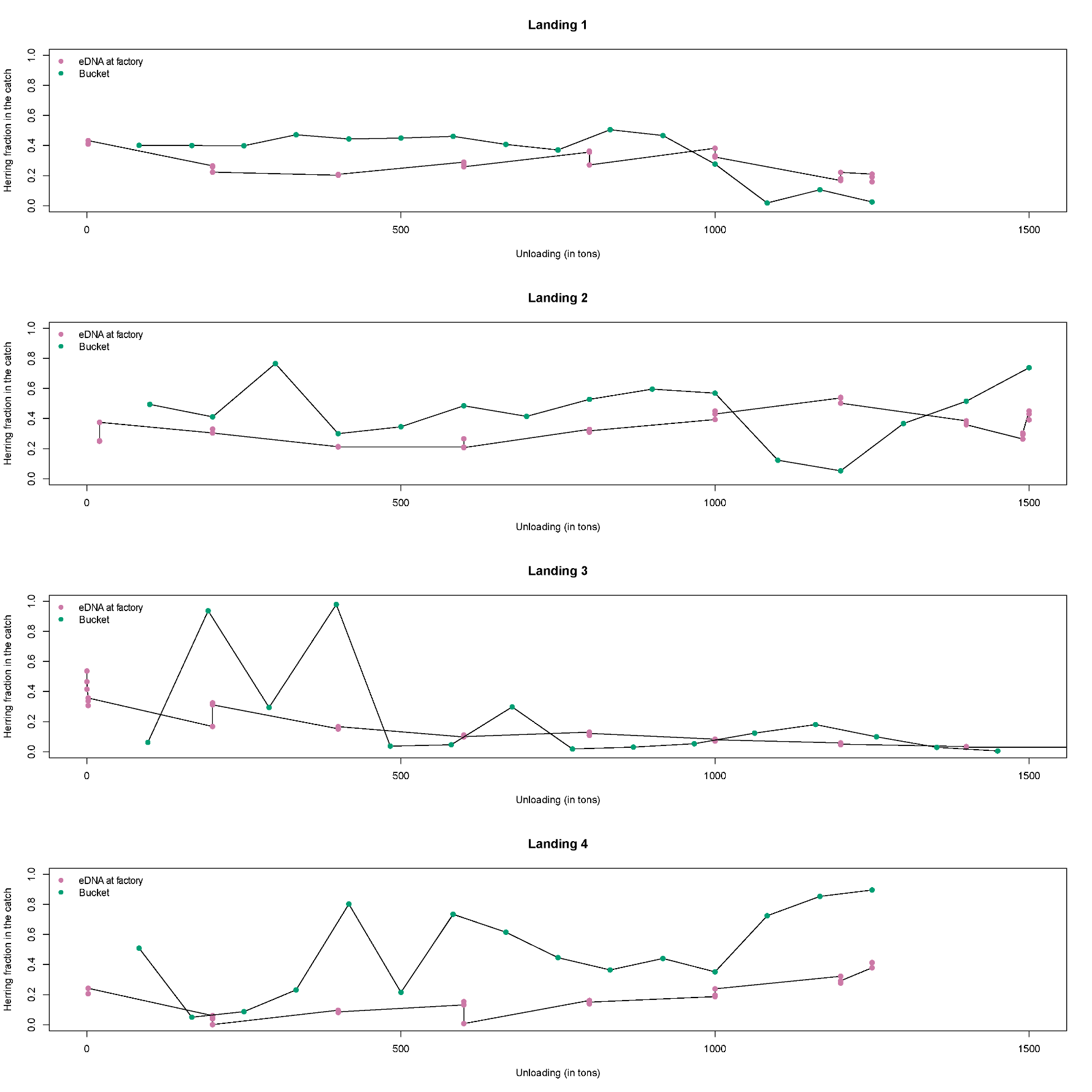


Supplementary Figure S10 Distribution of herring fraction throughout four different sprat landings (1-4). The herring fractions are represented as eDNA based fractions (pink) and fractions derived from subsamples collected for the bucket method.

Supplementary Table S11 In landing 4 the fisherman provided an additional estimation of the herring fractions for each tank filled with the sprat catch.

| Tank nr. | Estimated herring fraction |
| --- | --- |
| 1 | 0.96 |
| 2 | 0.2 |
| 3 | NA |
| 4 | 0.9 |
| 5 | 0.91 |
| 6 | 0.2 |

Supplementary Figure S12 Relationship between DNA-based herring fractions derived from both experiments and allometrically scaled weights (i.e. weights corrected for the differences in length of the fish, which affects DNA release into the environment).


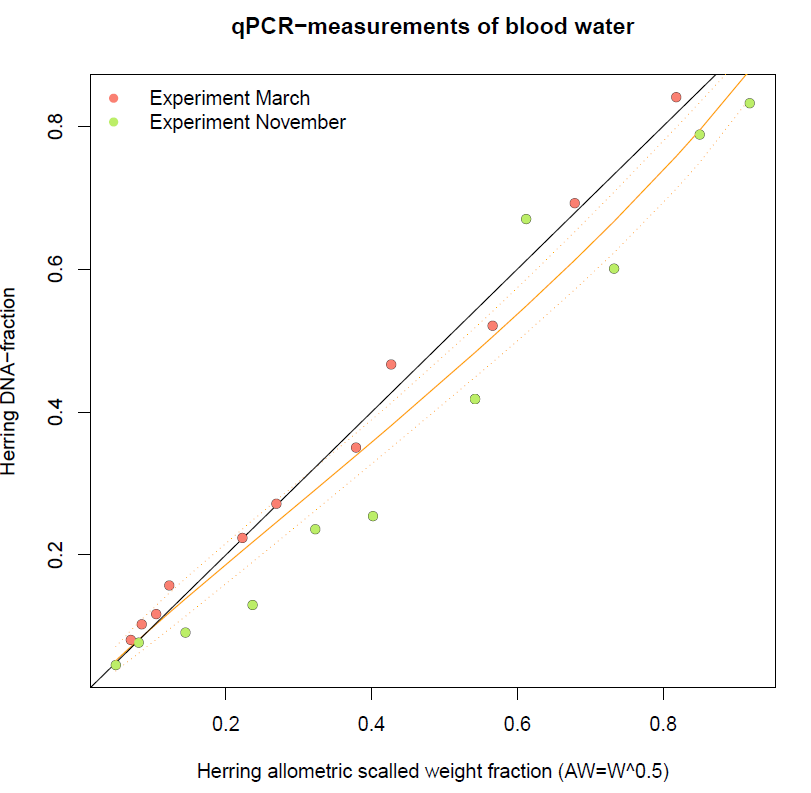

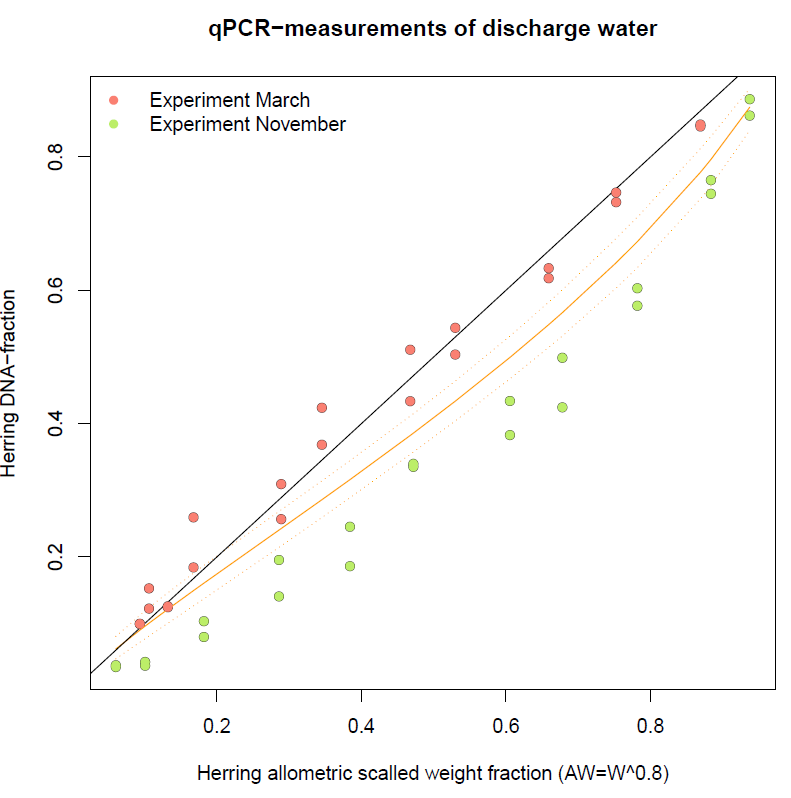


Supplementary Figure S13 herring bycatch fractions estimated using blood water collected from ship tanks. From each tank (i.e. tank name 1-8) three replicates were collected. No blood water was collected during landing 2.


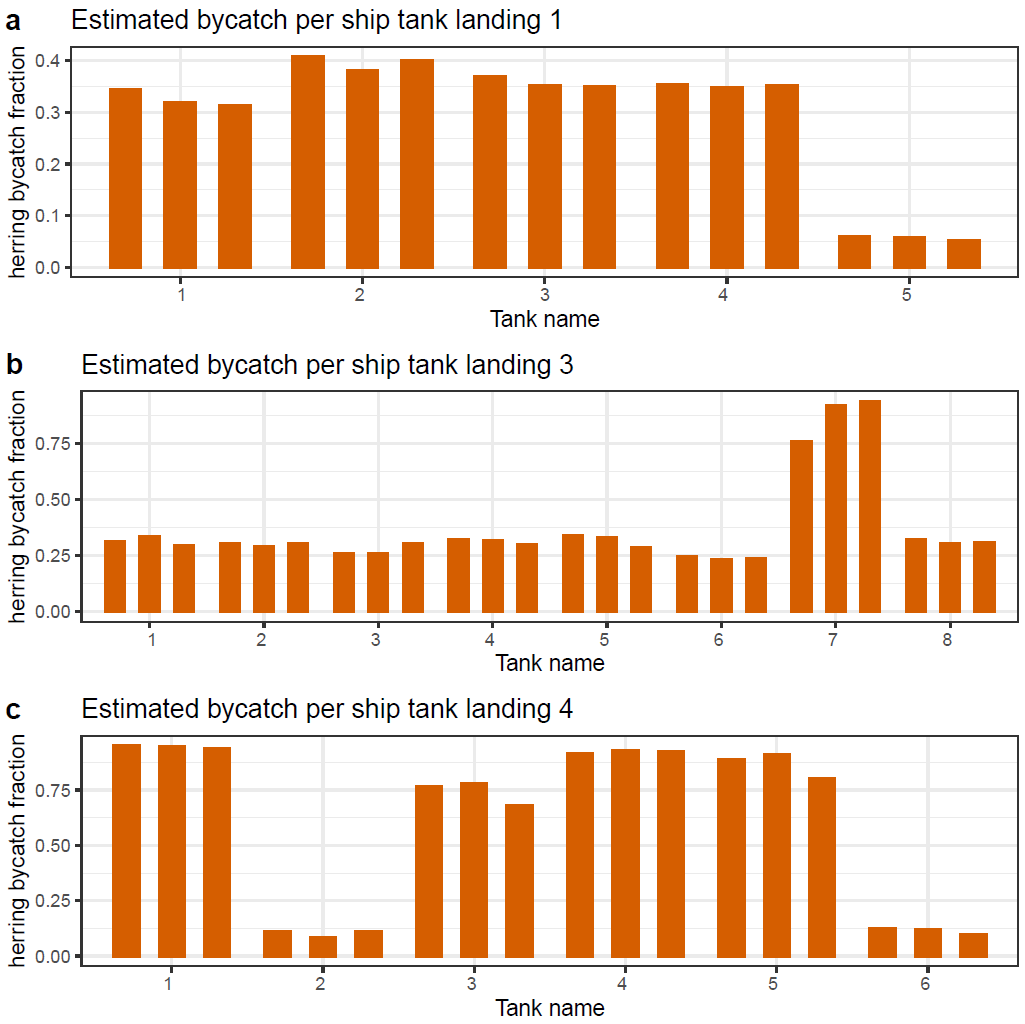


Supplementary Discussion S14

Unlike species-specific qPCR-based methods customized toward quantification of the target species, the DNA-metabarcoding approach is more universal, allowing the detection of many different species simultanously^1^ (Supplementary Fig. S2). The consequence of universality is increased chance for PCR drift, i.e. stochastic processes that affect the reproducibility of DNA metabarcoding^2,3^, likely causing high variation in the eDNA fractions in the replicates. To minimize PCR bias we used high PCR reaction volumes (20 µl)^3^ and high quality DNA samples^4^. The poor performance of the DNA-metabarcoding on ship production water could, however, also be caused by the presence of PCR inhibitors^5,6^.

1. Jacobsen, M. W., Hansen, B. K. & Nielsen, E. E. Possible uses of genetic methods in fisheries under the EU landing obligation. Eur. Land. Oblig. Reducing Discards Complex, Multi-Species Multi-Jurisdictional Fish. 407–427 (2018) doi:10.1007/978-3-030-03308-8_20/FIGURES/5.

2. Wagner, A. et al. Surveys of Gene Families Using Polymerase Chain Reaction: PCR Selection and PCR Drift. Syst. Biol. 43, 250–261 (1994).

3. Buchner, D., Beermann, J. A., Leese, F. & Weiss, M. Cooking small and large portions of “biodiversity-soup”: Miniaturized DNA metabarcoding PCRs perform as good as large-volume PCRs. Ecol. Evol. 11, 9092–9099 (2021).

4. Barba, D. M. et al. DNA metabarcoding multiplexing and validation of data accuracy for diet assessment: application to omnivorous diet. Mol. Ecol. Resour. 14, 306–323 (2013).

5. Shelton, A. O. et al. Toward quantitative metabarcoding. Ecology (2022) doi:10.1002/ECY.3906.

6. Schrader, C., Schielke, A., Ellerbroek, L. & Johne, R. PCR inhibitors – occurrence, properties and removal. J. Appl. Microbiol. 133, 1014–1026 (2012).
